# Anti-Obesity Effects of Medicinal and Edible Mushrooms

**DOI:** 10.3390/molecules23112880

**Published:** 2018-11-05

**Authors:** Kumar Ganesan, Baojun Xu

**Affiliations:** Food Science and Technology Program, Beijing Normal University—Hong Kong Baptist University United International College, 2000, Jintong Road, Tangjiawan, Zhuhai 519087, China; kumarg@hku.hk

**Keywords:** medicinal and edible mushrooms, cholesterol-lowering effects, antioxidants, anti-inflammatory, anti-obesity

## Abstract

Obesity is a group of metabolic disorders caused by multiple factors, including heredity, diet, lifestyle, societal determinants, environment, and infectious agents, which can all lead to the enhancement of storage body fat. Excess visceral fat mass in adipose tissue generate several metabolic disorders, including cardiovascular diseases with chronic inflammation based pathophysiology. The objective of the current review is to summarize the cellular mechanisms of obesity that attenuate by antioxidant potentials of medicinal and edible mushrooms. Studies have showed that mushrooms potentially have antioxidant capacities, which increase the antioxidant defense systems in cells. They boost anti-inflammatory actions and thereby protect against obesity-related hypertension and dyslipidemia. The practice of regular consumption of mushrooms is effective in the treatment of metabolic syndrome, including obesity, and thus could be a good candidate for use in future pharmaceutical or nutraceutical applications.

## 1. Introduction

Obesity is a group of disorders defined as a body mass index (BMI) of more than 30 kg/m^2^, in which enhancement of storage body fat deposited in the adipose tissue can cause deleterious health effects. The complications of obesity (e.g., diabetes, cardiovascular diseases (CVD), pulmonary diseases, obstructive sleep apnea, cancer, osteoarthritis etc.) are usually caused by a combination of high food intake, sedentary lifestyles, lack of physical activity and a genetic predisposition. Hence, obesity is a metabolic syndrome that reflects an imbalance between energy intake and expenditure [1,2]. It is measured by excess stored fat and high lipid content in the plasma. The quantity of total mass of fat is enhanced by the availability of adipocytes and proliferation and cell differentiation that results in both augmented number and size of fat cell [1]. Adipose tissue is a significant organ that contributes energy balance in the body. High-fat accumulation causes an unusual progress of white adipose tissue that contributes to obesity in humans [2]. The primary management of obesity involves diet, exercise, and surgical intervention. In addition, there are treatment strategies such as the prevention of high-calorie intake, suppression of appetite, and the therapeutic drugs which affect mobilization and consumption of lipids [3]. Nevertheless, a successful result is also confirmed only in a trivial number of the population. Therapeutic drugs for obesity, such as orlistat (Xenical^®^) and sibutramine (Reductil), are found to cause many complications that include emesis, insomnia, headache, myocardial infarction, stomach pain and constipation [4]. In the growing medical field, clinicians often face main complications of multiple risk factor syndrome, including diabetes, pulmonary diseases, osteoarticular diseases, CVD (atherosclerosis, elevated blood pressure, stroke, dyslipidemia) and some of the commonest forms of cancer [5]. These can lead to morbidity and mortality in which obesity is the foremost cause of these syndromes.

## 2. Etiology of Obesity

A combination of excess nutrients and a lack of physical activity is the primary causative factor in most cases of obesity. In addition, obesity is caused by hereditary, medications or mental illness and endocrine disorders [6]. Sometimes high proportions of obesity are seen at a communal level and persist due to readily accessible tastier food, changes the mode of transportation, and increasing urbanization [7]. Further studies have shown that some of the potential contributors to elevated obesity levels include hormonal disruptors, inadequate sleep, the variability of ambient temperature, smoking habits, cravings, usage of medications (e.g., antipsychotic drugs), pregnancy at a later age, inherited risk factors and elevated BMI [8,9].

### 2.1. Diet, Lifestyle, and Societal Determinants

The global rate of obesity increased more than threefold between 1980 and 2014. More than 600 million people were considered obese in 2014. Almost 40 percent of men and women 18 years old and above were overweight. Over 41 million children (<5 years old) were overweight or obese in Asia. It is predicted that 1.12 billion people will be obese around the world in 2030 [10]. In most of the developed countries people die due to their overweight condition and obesity. This scenario is also rising in lower-middle-income nations, mostly in urban backgrounds. Overweight or obesity rates in Africa (1990–2014) increased by nearly double from 5.4 million to 10.6 million [11]. Overweight is mostly caused by the consumption of energy-dense foods when the ingestion of carbohydrates is higher than in a fat diet [12]. In addition, the lack of physical activity highly influences the rate of enhancement of obesity. Presently, over ¾ of the global population is found to exercise inadequately, along with the increasing sedentary nature of daily tasks, laborsaving technology, altered modes of transportation, and increasing urbanization. There is an increasing relationship between television watching time and obesity threat in both adults and children [13]. WHO further specified global populations are being less active in leisure pursuits, environmental and societal alterations connected with the progress and deficiency of encouraging strategies in various sectors including education, health, urban planning, farming, transport, environment, food, and advertising [10]. In developed nation’s populations, those with higher incomes are more probable to have obesity than those with low income. No significant relation is seen between obesity and education in men. Nevertheless, higher income women have less obesity than low-income women. Furthermore, women with college degrees are less probable to have obesity compared with less educated women [12]. Smoking is another societal factor that influences overweight. Over ten years, those who quit smoking will increase in weight by 4.5 kg (men), and 5.0 kg (women), respectively [14].

### 2.2. Genetics

More than 70 percent of obesity is caused by heredity. This is a consequence of phenotypes, which are linked to adipose tissue distribution and excess body fat [2]. Excessive adiposity and increase with age are also influenced by heredity. According to Neel’s ‘thrifty gene’ hypothesis, genes that predispose to obesity in populations that often experienced hunger [15]. Individuals who possess these genes develop the ‘obesogenic’ environment and can become extremely obese. Nowadays, this is predominantly seen in Pima Indians and Pacific Islanders [15]. Many genes involved in glucose and lipid metabolism are subject to positive selection in Asian and African ethnic groups. The search for genes that enhance the vulnerability to improve obesity has become progressively significant. There have been several studies that have proved that the candidate genes are extremely associated with obesity and elevated body weight [11].

### 2.3. Medical and Psychiatric Illness

Numerous psychiatric prescriptions are recognized as weight gain agents that cause obesity in most psychiatric cases [16]. Weight gain is connected with the use of mood stabilizers (lithium, valproate), antipsychotics (clozapine and olanzapine), and antidepressants (amitriptyline), which may have serious long-term complications [17]. Medical sicknesses that threaten to augment obesity include congenital (hypothyroidism, dwarfism, cretinism) or genetic syndromes (Cohen syndrome), and night eating syndrome [18]. The menace of obesity is significantly higher in psychiatric patients than in patients with non-psychiatric complaints and these adverse effects of medications vary with age and sex [19].

### 2.4. Infectious Agents

The term “infectobesity” describes obesity of infectious origin. Infectious agents, especially viruses, have been identified to cause obesity in various animal models [20]. Human Adv36 can induce insulin sensitivity, obesity, and hepatic steatosis in chickens, mice, and monkeys. Canine distemper virus and avian adenovirus were reported to cause growth failure, fatty liver, and obesity in mice. An avian retrovirus (Rous-associated virus-7) in chicken, and Borna disease virus in rats caused neuronal degeneration in the brain, obesity, hyperuricemia, and dyslipidemia. Scrapie agents were also reported to induce obesity in mice and hamsters [20]. Infections may not be completely associated with obesity as a causal factor; however, there may be a connection to the consequences of obesity [21]. It has found that overweight individuals are highly vulnerable to several types of contagion, due to the weakening of the immune system. Moreover, in severe obesity cases, the course of infectious diseases is more severe [20].

## 3. Pathophysiology of Obesity

**Figure 1:**
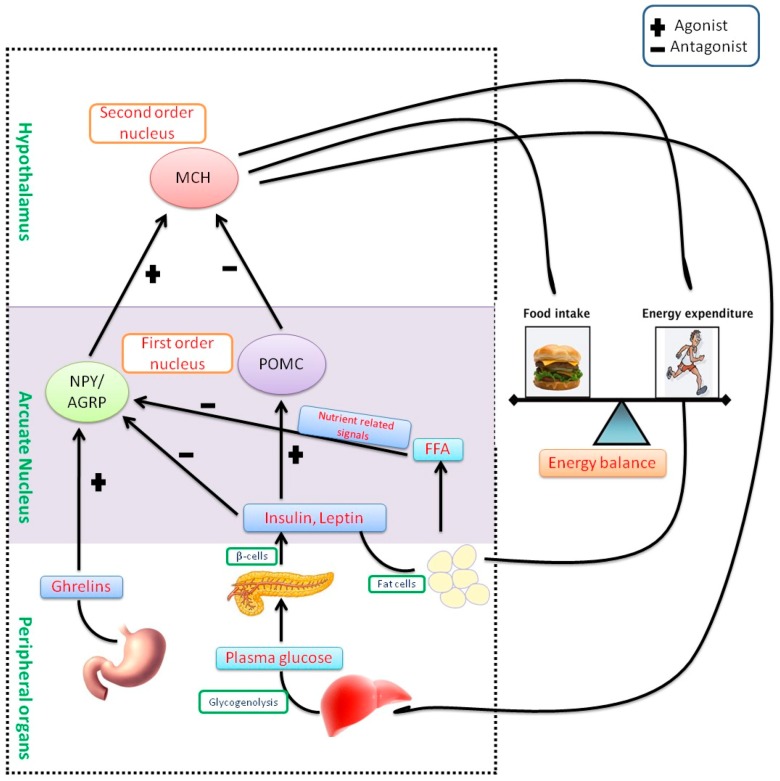
Pathophysiology of obesity and energy homeostasis—Melanin-concentrating hormone (MCH) is a peptide hormone, synthesized by the neurons of the hypothalamus, which normally stimulate food intake. These neurons are arbitrated by POMC as well as NPY/AGRP in an arcuate nucleus. Leptin and insulin are peptide hormones that activate POMC while inhibiting NPY/AGRP, consequently reduces body weight through energy expenditure or lipolysis and release free fatty acids (FFA). In contrast, the hunger hormone, ghrelin provokes NPY/AGRP and enhances body weight through the intake of food. MCH neurons are impeded by POMC cells, however, NPY/AGRP neurons are known to have an antagonist effect. Weight loss reduces insulin and leptin amounts in the blood while increasing ghrelin levels. This response is regulated by the arcuate nucleus (triggering of NPY/AGRP and impeding of POMC) that ultimately activates MCH neurons.

Hormones like leptin and ghrelin are internal mediators in humans involved in feeding and hunger. Leptin is a peptide hormone synthesized by adipocytes, which play a key role in the storage of fat in the body, and regulates long-standing appetite. When the energy reserves are adequate, leptin levels are increased and this would suppress further food ingestion. It is formed by the ‘*ob*’ gene in mice [22]. Ghrelin is another peptide hormone synthesized by the fundus lining of the stomach and epsilon cells of the pancreas, which regulates temporary appetite control. This peptide hormone plays a chief function in the maintenance of energy balance and body weight by impeding food ingestion and elevating energy expenditure through the hypothalamus [23]. Administration of leptin could be a powerful treatment in obese, mainly leptin deficient, individuals. A majority of obese persons are identified as leptin resistant and have been identified as having elevated leptin concentrations in their blood [24]. Leptin resistance in overweight individuals, which is due to the extreme synthesis of leptin with ineffective appetite control, is very common [22]. Although both leptin and ghrelin are synthesized peripherally, they regulate cravings through the hypothalamus, which regulates the intake of food and energy expenditure. The pro-opiomelanocortin (POMC) pathway in the hypothalamus is the significant circuit that stimulates satiety and inhibit feeding. It is initiated by an arcuate nucleus, lateral and ventromedial hypothalamus. The arcuate nucleus has another two functional neuron units, namely neuropeptide Y (NPY) and agouti-related peptide (AgRP) that provoke responses in the hypothalamus. Both induce feeding and inhibit satiety. Both units of the arcuate nucleus are mediated by leptin and ghrelin. These hormones inhibit the NPY/AgRP group while eliciting the POMC group and vice versa. Therefore, a deficiency of leptin signaling either via lacking leptin or leptin resistance encourages to overeating and ultimately causes some heritable and acquired types of obesity [22] (Figure 1).

## 4. Pathologies Associated with Obesity and Its Effects on Health

Obesity is an amplification of normal adiposity and is a central dogma in the pathophysiology of diabetes, cancer, dyslipidemia, hypertension, and atherosclerosis. It largely affects health because of the secretion of excessive adipokines [22,23]. Obesity is a key agent in metabolic malfunctions involving lipid and glucose metabolism and influences organ dysfunction involving the heart, liver, intestines, lungs, hormones, and reproductive functions. Obesity is coupled with numerous pathological effects due to the extra body weight (i.e., worsening of osteoarthritis, sleep apnea, gout, and pain of the vertebral column) [15,22]. Moreover, obesity is highly connected to the following occurrence and pathologies.

### 4.1. Chronic Inflammation and Endothelial Dysfunction

Over one billion adipocytes are present in humans, where they have the functions of storage of triglycerides in fat depots and supplying energy. In addition, they act as a major endocrine organ that regulates adipocyte hormones like leptin, adiponectin, and visfatin. Accompanying pancreatic hormones (insulin), these adipocyte hormones help normalize body-fat mass [25]. These body fat depots release inflammatory adipokines including cytokines (TNF-α, IL-1, and IL-6), complement proteins and growth factors, which generate local steatonecrosis in the vascular system and cause inflammation and endothelial dysfunction [25]. Studies have also demonstrated that the biomarkers of inflammation and endothelial dysfunction are connected with CVD, atherosclerosis, hypertension, and insulin resistance [26]. 

### 4.2. Hypertension and Atherosclerosis

**Figure 2:**
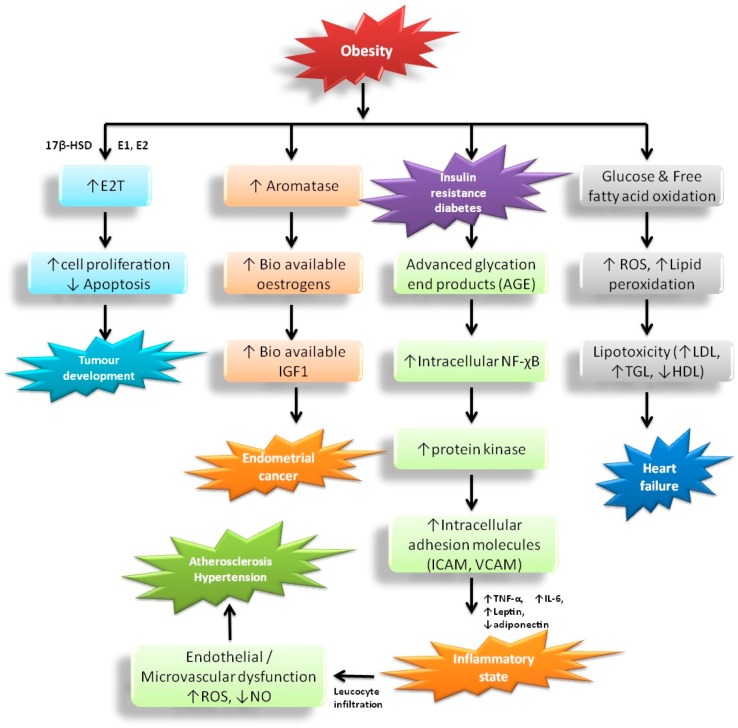
Physiological disorders related to obesity and its impact on human health. *Endometrial cancer*: Obesity elevates the threat of endometrial cancer via endocrine pathways. Elevated adiposity provokes aromatase action, leading to augmented estrogen in postmenopausal women. Estrogens generally elevate endometrial cell propagation and stimulating the production of IGF-binding protein 1 (IGF1)-cause endometrial cancer. *Tumor development*: Adipocyte normally synthesizes aromatase and 17β-hydroxysteroid dehydrogenase (17β-HSD). In obese persons, there is elevated transformation of the androgens Δ4-androstenedione (Δ4A) and testosterone (T) into the estrogens, oestrone (E1) and oestradiol (E2), respectively, by an enzyme, aromatase. 17β-HSD catalyze the Δ4A and E1 (less biologically active hormones) into the T and E2 (more active hormones), respectively. The circulating levels of sex-hormone-binding globulin aids to elevate the amounts of E2 and T that can readily diffuse across to target cells through binding with estrogen and androgen receptors. Ultimately, they inhibit apoptosis and promote cellular proliferation in the breast epithelium and endometrium. *Diabetes*: obesity leads to hyperinsulinemia and diabetes, which in turn produce AGE cause pro-inflammatory state by NF-χB, protein kinase, and intracellular adhesion molecules. Based on the reduction of NO and more leucocytes infiltration on the vessels cause endothelial and microvascular dysfunction, which influenced by oxidative stress (ROS), eventually cause atherosclerosis and hypertension. *Carbohydrate and lipid metabolism*: Excess glucose and fatty acid oxidation leads to lipid peroxidation, which facilitates lipoprotein toxicity and enhances the rate of hypertension in the blood vessels.

The incidence of hypertension and atherosclerosis are substantially higher in individuals with obesity (>60%) which affects different proportions of men (78%) and women (64%) [27]. The occurrence of hypertension enhances BMI dependence in both genders with increasing age [28]. Obesity affects individuals progressively, and later leads to morbidity and mortality. The incorporation of obesity, hypertension, and atherosclerosis has two key complications [29]. Initially, this combination is most dangerous for patients with obesity and high blood pressure that have elevated incidence of CVD, including coronary artery disease, carditis, cardiomyopathy, cardiac arrhythmia, end-stage renal disease, stroke, and obstructive sleep apnea [30]. Furthermore, obesity enhances the threat of treatment-resistant arterial blood pressure; as a result, several medications and equipment therapy like renal sympathetic denervation are needed [31]. Hormonal studies of the adipose tissue also found a connection between obesity, atherosclerosis, and hypertension, likely to cause adiposity dysfunction due to excess secretion of bioactive molecules and immunomodulators [32]. The impairment of adiposity in individuals with obesity causes insulin resistance, malfunctioning in the renin-angiotensin-aldosterone system as well as the sympathetic and parasympathetic nervous system [33]. These hormones are important to regulate the structure and functions of the kidney (Figure 2). 

Obesity can cause an upsurge in the risk of endometrial cancer through endocrine pathways. Obesity is related with augmented insulin levels, which may lead to elevated insulin-like growth factor 1 and synthesis of androgens that ultimately cause progesterone deficiency [27]. Lack of progesterone progresses to cause anovulation and consequently appears to be the most significant physiological risk factor for endometrial cancer in premenopausal women. Elevated adiposity normally provokes aromatase activity in postmenopausal women, leading to enhanced bioavailable oestrogen levels, endometrial cell propagation and stimulated production of IGF1 in endometrial tissue. After menopause (absence of exogenous oestrogen production), when ovarian progesterone synthesis has ceased completely, the more central risk factor appears to be obesity-related endometrial cancer development [28]. Adipocyte normally synthesizes aromatase along with 17β-HSD. In obese persons, there is elevated transformation of the androgens Δ4-Δ4A and T into the estrogens, E1 and E2, respectively, by an enzyme, aromatase. 17β-HSD catalyzes the conversion of Δ4A and E1 into T and E2 respectively. The circulating levels of sex-hormone-binding globulin helps elevate the amounts of E2 and T that can readily diffuse across to target cells through binding with estrogen and androgen receptors. Ultimately, they inhibit apoptosis and promote cellular proliferation in the breast epithelium and endometrium [29]. Obesity leads to hyperinsulinemia and diabetes, which in turn to produce AGE causing a pro-inflammatory state by NF-χB, protein kinase, and intracellular adhesion molecules. Based on the reduction of NO and more leucocyte infiltration in the vessels this causes endothelial and microvascular dysfunction, which are influenced by oxidative stress (ROS), eventually causing atherosclerosis and hypertension [30,32]. In addition, obesity elevates excess glucose and fatty acid oxidation levels that lead to lipid peroxidation and ROS generation, which facilitate lipoprotein toxicity and enhances the rate of hypertension and formation of plaque in blood vessels [27,28,29,30].

### 4.3. Dyslipidemia and Cardiac Alterations

Obesity is the most widespread cause of dyslipidemia, which produces metabolic syndrome [34]. Overproduction of lipids due to obesity and insulin resistance results in elevated TG storage in non-adipose tissues [35]. In addition, LDL rich in TG, partly degraded by a lipolytic enzyme (hepatic lipase), are transformed into smaller LDL, causing atherosclerosis [36]. Moreover, obesity enhances the threat of angina, cardiac arrest and death, and an unusual heartbeat. Elevated heartbeat in obese individuals increases the frequency of ventricular dysrhythmias and cardiac arrhythmias. The annual rate of cardiac deaths is almost 40 times higher in obese individuals than in non-obese persons [31].

### 4.4. Metabolic Syndrome

Obesity is the key danger factor for type 2 diabetes. In developed countries, obese individuals are ten times more likely to be identified with diabetes than persons of a healthy weight. At present, about 90% of individuals with diabetes are overweight or obese. Individuals with severe obesity are at greater risk of type 2 diabetes than an obese person with a lower BMI [34]. Rizvi [37] confirmed that the connection between obesity and diabetes, and impaired glucose intolerance. Generally, in obese persons, there is massive adipose tissue, which produces a huge quantity of glycerol, free fatty acids, pro-inflammatory cytokines, advanced glycation end products, intracellular adhesion molecules, and hormones [37]. These substances are highly connected with insulin resistance, which develops hyperinsulinemia with excess pancreatic islet stimuli and decreases or impairment of receptors leading to type 2 diabetes. Ganesan and Gani [38] stated that obesity is the most important component of the metabolic syndrome/disorder, described by the co-occurrence of impaired glucose tolerance, diabetes, insulin resistance, abdominal obesity, hypertension, and the combination of increase TG and decrease HDL cholesterol (Figure 2).

### 4.5. Cancer and Neurodegenerative Disorders

The World Cancer Research Fund and International Agency for Research on Cancer have suggested that overweight or obese individuals are highly susceptible to get various cancers, namely adenocarcinoma of the esophagus, colon, breast, endometrium, and kidney [39]. Epidemiological studies have also indicated that malignancies of the liver, gallbladder, and pancreas are obesity-associated and that obesity could enhance the danger for other malignancies including thyroid, prostate, leukemia, non-Hodgkin’s lymphoma, multiple myeloma, and melanoma [40]. It has been appraised that about 20% of all cancer demises in the United States can be recognized as connected with overweight and obesity [41,42]. The levels of circulating estrogens are strongly associated with adiposity. These obesity-related malignancies are mostly ascribed to the elevated levels of estrogen in the adipose tissue, inflammation, and infiltration of macrophages [43]. Overweight and obesity cause a mental and emotional state in a person that reduces self-worth to mental depression. Certainly, the incidence rates of anxiety and depression are 3–4 fold increased among obese people [44]. Obesity escalates considerably the menace of neurodegenerative disorders such as Alzheimer’s, multiple sclerosis, Parkinsonism, and amyotrophic lateral sclerosis. For incidence, a solid correlation occurs among BMI and increase concentrations of amyloid. This protein normally accumulates in the Alzheimer’s patient brain, eventually destroying nerve cells and creating mental and social problems [45].

### 4.6. Sex Hormone Imbalance

Obesity can influence reproductive functions causing problems such as hormonal imbalance, impairment in ovulation, and infertility in malea and females [43]. Obesity has typically been linked with impaired fecundity. Obese women are less likely to conceive per cycle. These women normally suffer distresses to the hypothalamic-pituitary axis, menses disruption and are 3–4 times more likely to get oligo-/anovulation [46]. Decreased levels of estrogen after menopause have also been linked with excess adiposity and accumulation of visceral fat [47]. Besides that, obesity induced by hormonal imbalances is connected to a range of adverse health complications as a result of cardiovascular abnormalities and insulin resistance [46]. It is well known that testosterone is a primary hormone in the pathophysiology of overweight and obesity. Decreased levels of testosterone are connected with high-fat mass, particularly central adiposity and decreased thin mass in males. These characteristic features are connected to metabolic syndrome and testosterone deficiency and are linked with energy inequity, hyperglycemia, insulin resistance and dyslipidemia [48]. Testis dysplasia and alteration of sex hormone levels exist in obese male adolescents. Obesity and accumulation of fat lead to elevated estradiol and declined total and free testosterone that leads to erectile dysfunction [49]. 

## 5. Treatment of Obesity

Lifestyle (diet, exercise/physical exercise), pharmacotherapy (weight loss drugs and hormone treatment), and behavioral therapy may cause modest weight reductions in severely obese individuals. Lifestyle with pharmacotherapy has been shown to induce a 2–10% weight loss in obese individuals per annum [50]. However, long-term treatment for obesity is medically a most challenging task.

### 5.1. Dietary Intervention and Diet Control

There is a great positive link between the quantity of total body fat and visceral adiposity. Hence, any dietary intervention that will diminish total adiposity is likely to induce some loss of abdominal fat. Lifestyle changes also promote weight loss, which can lead to reduced visceral and subcutaneous adipose tissue. The nutritional recommendation with an emphasis on a low-calorie and low-fat diet, with consumption of 800 to 1500 kcal of energy/day, persists. Reduced intake of calories (in the range of 500–1000 kcal) is likely to reduce excess body weight [26]. Their will permit approximately 1 to 2 pounds of weight reduction in a week. Fasting or starvation are also indicated as causes of weight loss in obesity [50]. Nutrition awareness is important for body weight management. 

### 5.2. Physical Activity and Pharmacotherapy 

Energy balance comprises the balance between intake of calories and the use of energy [51]. The main causes of obesity are due to the intake of easy and cheap readily available high-calorie fat diets combined with an inactive lifestyle. Hence, negative energy balance (eating less food and enhanced energy expenditure) is the only way to reduce obesity and overweight. Consistent physical activity is a key element to dropping weight. A diversity of physical exercises are helpful and easy to execute. Exercises not only help to reduce weight but also enhance cardio-respiratory fitness and prevent CVD [51]. Exercise is the most effective therapy for obesity together with a low calorie regime. Medication and weight loss surgery can help to reduce weight in obese persons. Medications regularly require long administration periods as many individuals regain their lost weight when medication is suspended. The suspension of medication use by individuals is typically due to their major side effects, cost, and a potential lack of insurance coverage [52]. 

### 5.3. Surgical Treatment

Weight loss surgery (WLS)/metabolic surgery is an effective treatment for obesity [53]. The National Institute of Health consensus has recommended surgery only for those obese individuals with high BMI (>35) and who have a serious clinical illness with sleep apnea.

### 5.4. Natural Products

Natural products that are possible medications for obesity are under investigation. According to Ayurvedic medicine, this can be an outstanding, effective, and safe strategy [54]. Various natural bioactive compounds can influence weight loss and avert diet-induced obesity. Hence, these products have been extensively consumed for the treatment of abdominal obesity and overweight [55,56,57]. Various studies have shown the role of natural metabolites obtained from medicinal plants, which are used in the prevention of obesity and obesity-related chronic disorders. This anti-obesity or weight loss mechanism may be influenced by the medicinal plants, which modulate appetite suppression or the restraint of lipid and carbohydrate metabolic enzymes or interfere with adipogenesis [58,59,60]. Recent animal investigations that have also confirmed the role of various phytochemical-based strategies encourages research into the prevention of obesity. These cellular studies support the notion that the consumption of dietary bioactive compounds decreases the proliferation of preadipocytes, reduces the viability and differentiation of adipocytes, promotes lipolysis and fatty acid β-oxidation, and minimizes triglyceride accumulation and inflammation [7,9]. Furthermore, animal studies have strongly indicateed that the regular consumption of dietary bioactive compounds has a significant influence on obesity, as demonstrated by the reduction of body weight and stored fat mass via increasing energy and fat burning, and regulating glucose hemostasis [61]. 

### 5.5. Mushrooms

Mushrooms have been extensively used as foods, nutraceuticals, and medicines since time immemorial [61]. They are recognized as one of the most important food supplements for their vital roles in human health, nutrition, and various illnesses. They contain various bioactive compounds, including primary metabolites that could avert oxidative stress [62]. Secondary metabolites such as polysaccharides (mainly β-d-glucans), heteroglycans, chitinous substances, peptidoglycans, proteoglycans, lectins, RNA components, lectins, lactones, alkaloids, terpenes, flavonoids, terpenoids, steroids, phenols, glycoproteins, nucleotides, fatty acids, vitamins, proteins, amino acids, antibiotics and minerals that have favorable impacts on the human body and protect it from the diseases [62]. These bioactive compounds are excellent antioxidants and anti-inflammatory agents beneficial to the CNS, heart, kidney, and liver [63]. Furthermore, it has been proven that these bioactive compounds act as chemopreventive agents and protect most serious diseases, including diabetes, obesity, CVD and neurodegenerative diseases [1]. 

Several mushrooms have been widely consumed in most of the developed and developing countries by different ethnicities, races and cultures and are proved to maintain normal health and prevent or treat dreaded illnesses [62]. Nutritional analyses of mushrooms found that edible mushrooms contain vital nutrients, taste, flavor and physiological functions [64]. They are rich in high quality proteins, polyunsaturated fatty acids (with a relatively low content of total fat), vitamins, minerals, and fiber. Mushrooms produce low energy which is favorable for weight loss; the contain low glucose, and high mannitol, that is exactly appropriate for diabetics; and have no cholesterol and low sodium, which is good for people suffering from hypertension [63,64]. Furthermore, mushrooms have a high content of vitamin D and B-complex with a high content of minerals and a significant quantity of many trace elements, especially of selenium, which is a potent antioxidant [65]. In addition to their nutritive value, edible mushrooms have exclusive features in terms of color, palate, flavor, odor, and texture that make them more attractive for human ingestion. Several studies have recommended regular ingestion of certain mushrooms are either as a regular food or as extracted compound (nutraceuticals). Some of these compounds (polysaccharides) are active in both preventing and treating various diseases [64].

Dietary and medicinal mushrooms are widely recognized for their immunomodulatory, hepatoprotective, antiviral, antinociceptive, antitumor, anticancer, antidiabetic, and antimicrobial properties [66,67]. Mushrooms constitute 22,000 well-known species, extensively present on Earth and about 10% of them have been investigated [27]. The dietary mushrooms that have unique functional and medicinal features include *Lentinus*, *Auricularia*, *Hericium*, *Grifola*, *Flammulina*, *Pleurotus*, and *Tremella* species. Medicinal mushrooms recognized for their medicinal properties include *Ganoderma*, *Trametes*, etc. [65]. The beneficial effects of edible mushrooms and their polysaccharides on the gut microbiota [68,69] that is highly associated with obesity and diabetes is presently a dynamic niche research area [70]. A study in mice showed that the extracts of G. lucidum decrease overweight by modulating the microbiota, and hence mushrooms could be a novel prebiotic to control obesity [68]. The impact of a high-fat diet (HFD) on the microbiota in the gut is more complex than the impact on energy equilibrium. Studies showed that HFD-induced alterations in gut microbiota provide a reduction of *Bacteroides* and *Firmicute* elevation, which are linked to high energy harvest, fat storage and eventually gut inflammation and permeability [70]. Mushrooms help to regulate dysbiosis and augment antiobesity effects. Holmes [71], and Chang et al. [69] indicated that *G. lucidum* decreases obesity in mice by regulating the composition of the microbiota. These considerations further suggest that the likely functions of microbiota in the polysaccharide-induced reduction of obesity and diabetes. Furthermore, modulating microbiota with the consumption of mushroom could also help maintain glucose homeostasis and reduce insulin resistance linked to diabetes and obesity. Huang et al. [72] demonstrated that the polysaccharides obtained from *Pleurotus tuber-regium* mushrooms showed antihyperglycemic and antihyperlipidemic potential and reduced oxidative stress in obese diabetic rats. 

## 6. Anti-Obesity Effects of Edible and Medicinal Mushrooms

Mushrooms have high nutritious value with numerous bioactive compounds that have well-known impacts on various cardiac markers [73,74,75,76,77,78,79,80,81,82]. Mushrooms have been well documented in traditional medicine as having hypocholesterolemic effects. These effects are connected to CVD related lipid metabolism, anti-inflammatory properties, and the prevention of oxidative stress with platelet agglutination [74]. Furthermore, the consumption of mushrooms diminishes CVD and obesity due to their significant amounts of bioactive compounds [8]. Studies further showed that the cholesterol-lowering effect might be due to a decrease in VLDL [73] and decrease in the catalytic functions of HMG-CoA reductase and amplification of the rate of cholesterol catabolism [75]. A study evidenced that mushrooms reduced TG, TC, plasma glucose and hypertension in diabetic rats [73]. These results further suggested that the intake of mushrooms provides health aids by acting on the atherogenic profile under hyper- and normocholesterolemic circumstances in rats [73]. 

Obesity unfavorably affects systemic immunity via the inflammatory index, and platelet markers [76]. Numerous investigations have examined the anti-obesity effect of polysaccharides obtained from different mushrooms in vitro as well as in vivo. Polysaccharide obtained from *Coriolus versicolor* triggered mice splenocytes via the MAPK-NF-κB signaling pathway that induces an immunomodulatory effect [77]. A polysaccharide obtained from *Tremella fuciformis* prevented the variation of 3T3-L1 adipocytes by decreasing the expression of mRNA suggesting the possible significance of the polysaccharide as an anti-obesity prebiotic [63]. Treatment of adipocytes with *G. lucidum* reduced adipogenic transcription factor expression that stimulates transportation, storage of glucose and lipids, and activates AMPK signaling pathways suggesting the potential significance of the polysaccharide as an antiobesity and antidiabetic agent [78]. Long-term (1 year) and short-term (4-day) clinical studies with obese or diabetic participants asked them to evaluate the impact of substituting 20% of high-energy beef with 20% of low-energy white button mushrooms in the diet [79,80]. The results showed that the mushroom regime consumers had lesser BMI, decreased belly circumference, and increase satiety without diminishing palatability. The authors of the studies concluded that the consumption of white button mushrooms (*Agaricus bisporus*) has potential as an antidiabetic and antiobesity. Similarly this work has been extended to include other extremely health-promoting mushroom varieties, like *Hericium erinaceus* (Lion’s mane) and *Lentinus edodes* (shiitake) species [81,82]. The in vitro and in vivo actions of edible and medicinal mushrooms and its anti-obesity potentials are summarized in Table 1.

### 6.1. Actions on Hypertension

Hypertension is regulated by several mechanisms, one of the most significant of which is a renin-angiotensin-aldosterone system (RAS). Normally RAS is regulated by the Zn-metallopeptidase enzyme, angiotensin-converting enzyme (ACE). The main function of ACE is involvement in the transformation of an Ang-I decapeptide into an Ang-II octapeptide. Typically Ang-II (a potent vasoconstrictor), cooperates with the Ang-II type 1 receptor (AT1) provoking the synthesis of aldosterone, which elevates sodium and water retention in the kidney and correspondingly raises blood pressure by enhancing the volume of the intravascular fluid [83]. 

**Figure 3:**
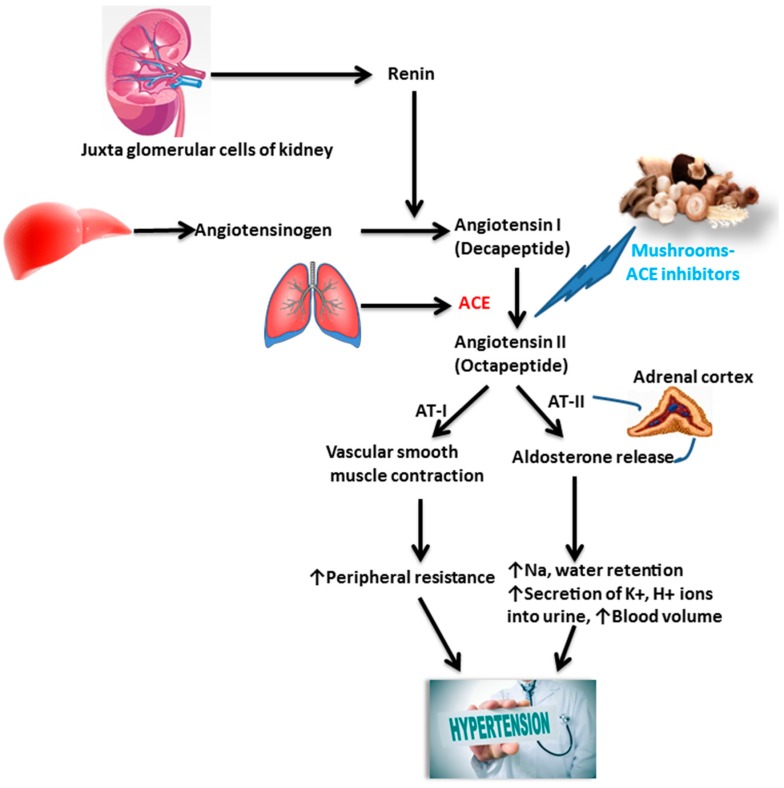
Antihypertensive effect of mushrooms—Mushrooms are found to be Angiotensin Converting Enzyme (ACE) inhibitors, which help to reduce hypertension by constraining ACE. This enzyme is accountable for transforming the inactive protein Ang I into the active Ang II. The renin-angiotensin system–angiotensin II has a multifaceted range of effects on the maintenance of blood pressure and this impact elevates sodium and water retention through the release of aldosterone.

ACE inhibitors are used as an effective prescription for the inhibition and treatment of hypertension-related diseases [29]. Commercially available ACE inhibitors are chemically synthesized and clinically used as antihypertensive drugs [84]. The usage of these synthetic ACE inhibitors has side effects such as a cough, dysgeusia and various hypersensitivity reactions. Therefore, it would be useful to develop and use safe and comparatively low-cost ACE inhibitors of natural origin (Figure 3). ACE inhibitors have been reported to diminish mortality in patients with hypertension [85]. Recently, investigators have described that *Pleurotus ostreatus* [86], *P. cystidiosus* [85], *P. cornucopiae* [87], *Auricularia auricula-judae* [88], *Ganoderma leucocontextum* [65], *Grifola frondosa* [89], *Agaricus bisporus* [30] and *Leucopaxillus tricolor* [29] are all ACE inhibitors that decrease hypertension. Wild mushrooms in Nepal that also have ACE inhibitor activities were tested by Bang et al. [90]. 

### 6.2. Actions on Dyslipidemia

**Figure 4:**
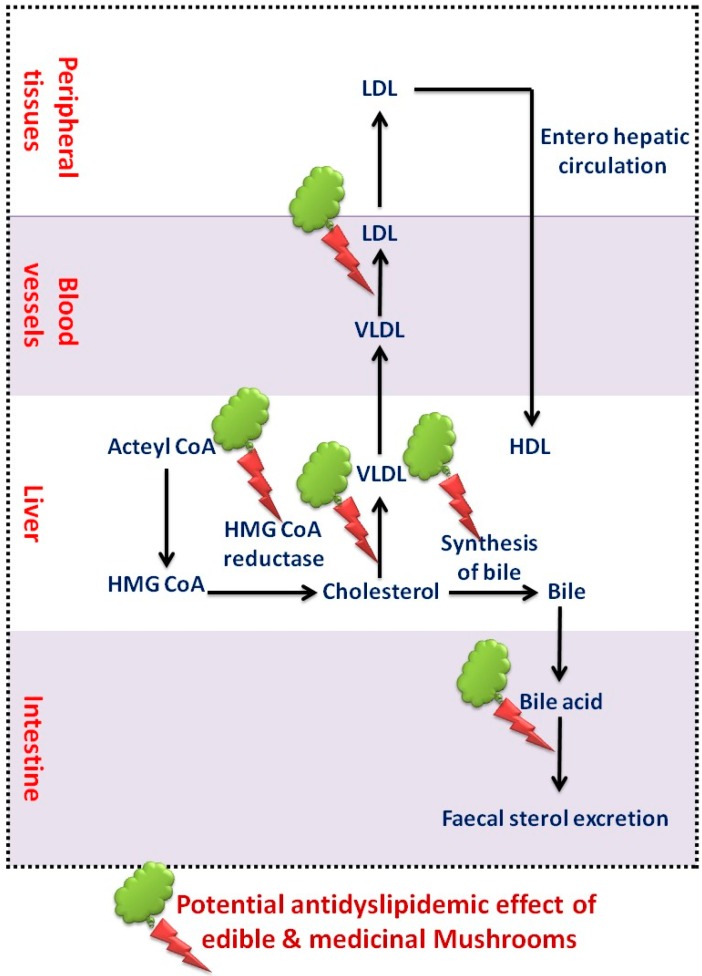
Effect of edible and medicinal mushrooms on dyslipidemia.

Edible mushrooms are rich in dietary fiber, vitamins, proteins, microelements, and low in fat that, making them the ultimate diet for treating atherosclerotic plaque [88]. Mushrooms prevented weight gain in a rodent study suggesting a precious treatment for obesity and greater significance in the prevention of hyperlipidemia and CVD [1]. *Pleurotus ostreatus* is an edible mushroom, which decreases TC, TG, blood glucose and BP in diabetic individuals. These outcomes suggested that ingestion of *P. ostreatus* offers greater health benefits by acting on the atherogenic lipid system [75]. Edible white button mushroom (*Agaricus blazei*), *Kluyveromyces marxianus* [91], *P. ostreatus* [92] and *Auricularia polytricha* [93] are found to be well-known lipid-lowering agents by competitively preventing HMG-CoA reductase activity, which plays a vital role in cholesterol biosynthesis [35]. The results of *P. ostreatus* on serum TG levels could be enlightened due to increased lipoprotein lipase activity by increasing lipase mRNA expression [75] and suppression of diacylglycerol acyl-transferase, which catalyzes the final step in TG biosynthesis in rat liver microsomes [74]. The mechanisms associated with cholesterol biosynthesis implicated in the hypocholesterolemic impact of mushrooms are represented in Figure 4. An adequate level of PUFAs is found in edible mushrooms, which facilitates the reduction of serum cholesterol [91]. As they lacking the *trans*-isomers of unsaturated fatty acids, mushrooms can elevate the serum TC to HDL that reduces the cardiovascular menace [74]. Intake of dietary fiber may also affect serum lipid levels, decrease TC, LDL concentrations and eventually reduce CVD [65]. Mushrooms contain glucan-like viscous gels that prevent the TC and TG absorption. These sticky consistencies are highly associated with augmentation of fecal bile acids and SCFA excretion, which prevents acetate integration (a precursor of sterols and synthesis of fatty acids) to become serum lipids. *Auricularia auricula* and *Tremella fuciformis* possess high fiber content, which produces a lowering of LDL cholesterol levels and prevents CVD [88]. These findings might be the result of prevention of TG synthesis by enhancing the SCFA production through the dietary fiber fermentation by gut microbiota [72].

## 7. Conclusions and Future Perspectives

Obesity is a dreaded disease that affects a great proportion of the global population and contributes to extensive morbidity and death. Weight management is a lifelong progression as enduring weight decline is very hard to attain. The main cause of obesity is a disproportion between calorie intake and energy outlay resulting from multifaceted relations between hereditary and environmental influences. Effective weight control programs are urgently required to stabilize calorie intake with energy expenditure. Diet and physical activity can usually regulate weight control. Mushrooms are highly nutritive species containing enormous amounts of bioactive compounds (polysaccharides, fibers, terpenes, polyphenols, sterols, flavonoids, and alkaloids) that are potentially antioxidant-rich constituents with effects on numerous cardiac biomarkers to treat obesity-related cardiovascular system illnesses. Various animal studies have demonstrated that regular consumption of mushrooms significantly reduces hypertension, atherosclerosis, dyslipidemia, inflammation, and obesity. Nevertheless, this practice ought to be combined with regular physical exercise, as well as dietary and lifestyle alterations. The practice of regular consumption of mushroom might however result in synergistic and improved effects.

## Figures and Tables

**Table 1:** In vitro and in vivo actions of edible and medicinal mushrooms and their anti-obesity potential.

Edible/Medicinal Mushroom	Botanical Name	Study Model/Methods	Bioactive Compounds	References
Edible	*Agaricus campestris*	Hypercholesterolemic diet and STZ induced rats-plasma glucose, TG, TC, ALT, AST and LDL	Vitamin C, D, B_12_, folates, and polyphenols	[63]
Edible	*Agaricus bisporus*	High-fat diet in rats-serum cholesterol and hepatic LDL receptor mRNA	Fibers	[32]
		Hypertensive rats-Angiotensin I-Converting Enzyme assay	Oligopeptide	[30]
Edible	*Agaricus brasiliensis*	STZ-induced diabetic rats-plasma glucose, TG, TC, glycated hemoglobin, TBARS	Polyphenols and flavonoids	[94,95]
		Hypertensive rats-Angiotensin I-Converting Enzyme assay	Oligopeptide	[27]
Edible	*Boletus bicolor*	Hypertensive rats-Angiotensin I-Converting Enzyme assay	Oligopeptide	[96]
Edible	*Leucopaxillus tricolor*	Hypertensive rats-Angiotensin I-Converting Enzyme assay	Oligopeptide	[29]
Edible	*Catathelasma ventricosum*	STZ induced diabetic rats-plasma glucose, total TC, TG	Heteropolysaccharide	[97]
Edible	*Pleurotus geesteranus*	STZ induced diabetic rats-plasma glucose, total TC, TG	Polysaccharides	[98]
Edible	*Flammulina velutipes*	STZ-induced diabetic rats-SOD, GSH-Px, CAT, MDA, ALT, AST, BUN, CRE, TC, LDL-C and HDL-C	Polysaccharides	[99]
		In vitro-DPPH free radical & Hydroxy radical scavenging, in vitro α-glycosidase, aldose reductase inhibitory assays	Polysaccharides	[100]
Edible	*G. lucidum*	Hypertensive rats-ACE assay	Oligopeptide	[101]
Edible	*Gloeostereum incarnatum*	Hypertensive rats-ACE assay	Oligopeptide	[29]
Edible	*Grifola frondosa*	Hypertensive rats-ACE assay	Oligopeptide	[29]
		Hypertensive rats-ACE assay	Oligopeptide	[102]
Edible	*Hericium erinaceus*	C57BL/6J mice model-serum and hepatic TG levels	Flavonoids	[103]
		Hyperlipidemic rats-plasma total cholesterol, LDL, HDL, cholesterol, triglyceride, phospholipid, atherogenic index, and hepatic HMG-CoA reductase	Exo-polymer	[104]
Edible	*Hypsizygus marmoreus*	Hypertensive rats-ACE assay	Oligopeptide	[84]
Edible	*Lactarius deterrimus*	STZ-induced diabetic rats-plasma glucose, TG, glycated hemoglobin, glycated serum protein, and AGE, SOD, CAT, GSH levels	Polyphenols and flavonoids	[105]
Edible	*Lentinula edodes*	High-fat diet in rats-TG, TC, LDL, cholesterol 7-α-hydroxylase 1	Lentinan KS-2	[106]
		High-fat diet in rats-TG, TC, LDL, total lipids, phospholipids, LDL/HDL ratio, BIL, CRE, Urea, BUN, Uric acid, Total protein, Na, Ca, Cl, K, albumin, P, Mg	Lentinan KS-2	[107]
		High-fat diet in rabbits-TC, histological, immunohistochemical and morphometrically analysis	Lentinan KS-2	[8]
		High-fat diet in rats-TG, TC, ALT, AST, Urea, glucose, malondialdehyde	Lentinan KS-2	[108]
Edible	*Lentinus lepideus*	High-fat diet in rats-TG, TC, LDL, total lipids, phospholipids, LDL/HDL ratio, BIL, CRE, Urea, BUN, Uric acid, Total protein, Na, Ca, Cl, K, albumin, P, Mg	Lentinan KS-2, flavonoids	[109]
Edible	*Lenzites elegans*	in vitro enzymatic starch digestion assay	Polyphenols and flavonoids	[110]
Edible	*Morchella vulgaris*	Hypertensive rats-ACE assay	Oligopeptide	[29]
Edible	*Oudemansiella radicata*	Hypertensive rats-ACE assay	Oligopeptide	[29]
Edible	*Pholiota adipose*	Hypertensive rats-ACE assay	Oligopeptide	[111]
Edible	*Pholiota nameko SW-02*	mice hyperlipidemic models-blood lipid levels (TC, TG, HDL-C, LDL-C, and VLDL-C), liver lipid levels (TC and TG) and antioxidant status (SOD, T-AOC, MDA, and LPO)	Mycelia zinc polysaccharide	[112]
Edible	*Pleurotus abalonus*	Diabetic mice-Inhibition of the proliferation of hepatoma HepG2 cells and breast cancer MCF7 cells, antioxidant activity in erythrocyte hemolysis, blood glucose and TG	Polysaccharide-peptide complex LB-1b	[113]
Edible	*Pleurotus cornucopiae*	Hypertensive rats-Angiotensin I-Converting Enzyme assay	Oligopeptide	[87]
Edible	*Pleurotus cystidiosus O.K. Miller*	Hypertensive rats-Angiotensin I-Converting Enzyme assay	Oligopeptide	[85]
Edible	*Pleurotus djamor*	STZ-induced diabetic rats-SOD, GSH-Px, CAT, MDA, ALT, AST, BUN, CRE, TC, LDL-C and HDL-C	Mycelium zinc polysaccharides	[114]
Edible	*Pleurotus eryngii*	High-fat diet in rats-TG, TC, LDL, total lipids, phospholipids, LDL/HDL ratio, BIL, CRE, Urea, BUN, Uric acid, Total protein, Na, Ca, Cl, K, albumin, P, Mg	Polysaccharides	[115]
Edible	*Pleurotus ferulae*	High-fat diet in rats-TG, TC, LDL, total lipids, phospholipids, LDL/HDL ratio, BIL, CRE, Urea, BUN, Uric acid, Total protein, Na, Ca, Cl, K, albumin, P, Mg	Polysaccharides	[116]
Edible	*Pleurotus ostreatus*	High-fat diet in rats-TG, TC, LDL, total lipids, phospholipids, LDL/HDL ratio, BIL, CRE, Urea, BUN, Uric acid, Total protein, Na, Ca, Cl, K, albumin, P, Mg	Polysaccharides	[86]
Edible	*Pleurotus pulmonarius*	Hypertensive rats-Angiotensin I-Converting Enzyme assay	Oligopeptide	[117]
Edible	*Pleurotus salmoneostramineus* L. *Vass*	High-fat diet in rats-TG, TC, LDL, total lipids, phospholipids, LDL/HDL ratio, BIL, CRE, Urea, BUN, Uric acid, Total protein, Na, Ca, Cl, K, albumin, P, Mg	Polysaccharides	[118]
Edible	*Pleurotus tuber-regium*	Ob diabetic rats-TC, TG, LDL, HDL, and PPAR-α mRNA expression	Polysaccharides	[72]
Edible	*Ramaria botrytoides*	Hypertensive rats-ACE assay	Oligopeptide	[29]
Edible	*Russula aeruginea*	Hypertensive rats-ACE assay	Oligopeptide	[29]
Edible	*Tremella fuciformis*	in vitro α-glycosidase, aldose reductase inhibitory assays, DPPH free radical scavenging	Polyphenols and flavonoids	[119]
Edible	*Tricholoma giganteum*	Hypertensive rats-ACE assay	Oligopeptide	[120]
Edible	*Tricholoma matsutake*	Hypertensive rats-ACE assay	Oligopeptide	[96]
Edible	*Tuber micheli*	Hypertensive rats-ACE assay	Oligopeptide	[29]
Edible	*Pleurotus ostreatus*	TC content in serum, lipoproteins in the liver, and HMG-CoA reductase in liver microsomes	Polysaccharides	[121]
		Inhibition of HMG CoA reductase-lovastatin	Polysaccharides	[92]
Edible	*Adiantum capillus-veneris* L.	High cholesterol diet fed Wistar rats-Pancreatic triacylglycerol lipase and α-amylase/α-glucosidase, OGTT, TC, TG	Polyphenols	[9]
Edible	*Aster spathulifolius* Maxim	High-fat diet fed Wistar rats-body weight gain, visceral fat pad weights, serum lipid levels, as well as hepatic lipid levels, numbers of lipid droplets, expression of fat intake-related gene ACC2 and lipogenesis-related genes (e.g., SREBP-1c, ACC1, FAS, SCD1, GPATR, AGPAT, and DGAT), fatty acid oxidation and thermogenesis-related genes (e.g., PPAR-α, ACO, CPT1, UCP2, and UCP3), phosphorylated AMPKα, phosphorylated ACC	Polysaccharides	[3]
Edible	*Kluyveromyces marxianus*	The high-fat diet fed Wistar rats-TC, TG, HDL-C, LDL-C, levels in the serum and liver, atherogenic index	Polysaccharides	[91]
Edible and Medicinal	*Collybia peronata*, *Ganoderma australe*, *Ganoderma lingzhi*, *Heterobasidion linzhiense*, *Heterobasidion linzhiense, Inocybe* sp., *Inonotus andersonii, Lactarius hatsudake, Lenzites betulina, Panellus* sp., *Phellinus conchatus*, *Phellinus gilvus*, *Phlebia tremellosa*, *Postia stiptica, Rigidoporus* sp., *Trametes versicolor*, *Tricholoma caligatum*	ACE Inhibitory Assay	Polyphenol	[90]
Medicinal	*Armillariella mellea*	STZ-induced diabetic rats-plasma glucose, TG, TC, ALT, AST	exo-biopolymers	[122]
Medicinal	*Auricularia auricula-judae*	High-fat diet in mice-phospholipids, liver enzymes, TG, glycerol, glycerol-3-phosphate dehydrogenase	Phenolic compound	[88]
Medicinal	*Collybia confluens*	STZ induced animal model-plasma glucose, total TC, TG, ALT, AST	Exo-polymer	[123]
Medicinal	*Cordyceps militaris*	In vitro-Superoxide anion, DPPH free radical & Hydroxy radical scavenging, In vitro-HMG-CoA reductase and α-glucosidase	Polysaccharides	[124]
		mice hyperlipidemic models-blood lipid levels (TC, TG, HDL-C, LDL-C, and VLDL-C), liver lipid levels (TC and TG) and antioxidant status (SOD, T-AOC, MDA, and LPO)	Polysaccharides	[73]
Medicinal	*Cordyceps sinensis*	STZ-induced diabetic rats-plasma glucose, TG, TC, ALT, AST	exo-biopolymers	[122]
Medicinal	*Coriolus versicolor*	STZ-induced diabetic rats-plasma glucose, TG, TC, ALT, AST	exo-biopolymers	[122]
Medicinal	*Flammulina velutipes*	High-fat diet in rats-TC, LDL, body weight, food intake, liver weight, cecum weight, cecum pH, Cecal acetic acid, butyric acid, and total SCFA	Fibers	[89]
		High-fat diet in male hamsters-TG, TC, LDL, total lipids, phospholipids, LDL/HDL ratio	Dietary fiber, polysaccharide, and mycosterol,	[125]
Medicinal	*Fomes fomentarius*	STZ-induced diabetic rats-plasma glucose, TG, TC, ALT, AST	Exo-biopolymers	[89]
Medicinal	*Ganoderma leucocontextum*	In vitro-HMG-CoA reductase and α-glucosidase	Lanostane (Triterpenes)	[65]
Medicinal	*Ganoderma lucidum*	STZ-induced diabetic rats-plasma glucose, TC, TG, glycated hemoglobin, TBARS	Polysaccharides	[94,95]
		STZ-induced diabetic rats-plasma glucose, TG, TC, NO, SOD, CAT, GPx	Polysaccharides	[126,127,128]
		in vitro α-glycosidase, aldose reductase inhibitory assays, DPPH free radical scavenging	Polyphenols and flavonoids	[119]
Medicinal	*Ganoderma philippii*	in vitro enzymatic starch digestion assay	Appanoxidic acid A	[110]
Medicinal	*Grifola frondosa*	High-fat diet in rats-TC, LDL, body weight, food intake, liver weight, cecum weight, cecum pH, Cecal acetic acid, butyric acid, and total short-chain fatty acid	Fiber	[89]
Medicinal	*Lentinus edodes*	STZ induced animal model-plasma glucose, total cholesterol, and triglyceride	Exo-polymer	[129]
		High-fat diet in rats-TC, LDL, body weight, food intake, liver weight, cecum weight, cecum pH, Cecal acetic acid, butyric acid, and total short-chain fatty acid	Fibers	[89]
Medicinal	*Paecilomyces japonica*	STZ-induced diabetic rats-plasma glucose, TG, TC, ALT, AST	exo-biopolymers	[122]
Medicinal	*Phellinus baumii*	STZ induced diabetic rats-plasma glucose, TG, TC, ALT, AST	Heteropolysaccharides and two proteoglycans	[130]
Medicinal	*Phellinus rimosus*	Alloxan-induced diabetic rats-plasma glucose, OGTT, TC, TG, SOD, CAT, GPx, and GSH	Polysaccharides	[131]
		STZ-induced diabetic rats-plasma glucose, lipid profile, ALT, AST, serum insulin, liver glycogen	Polysaccharides	[132]
Medicinal	*Rigidoporus ulmarius*	in vitro enzymatic starch digestion assay	Polysaccharides	[110]
Medicinal	*Tremella fuciformis*	ob/ob mice-Plasma glucose, OGTT, TG	Exopolysaccarides	[133]
		In vitro-ABTS radical scavenging activity, DPPH radical scavenging activity, LDL oxidation; NO synthase expression in RAW 264.7 cells	Polyphenols and flavonoids	[134]
Medicinal	*Ganoderma applanatum* and *Collybia confluens*	STZ-induced diabetic rats-Plasma glucose, TC, TG	Exo-polymer	[135]
Medicinal	*Auricularia polytricha*	Serum total lipids and TC	Polysaccharides	[93]
Medicinal	*Pleurotus sajor-caju* (Fr.) Singer	C57BL/6J mice fed on a high-fat diet-body weight, serum lipid, and liver enzymes, protein carbonyl and lipid hydroperoxide levels, enzymic antioxidants (SOD, CAT, and GPx) activities, Expression of hormone-sensitive lipase, adipose triglyceride lipase, peroxisome proliferator-activated receptor gamma, sterol regulatory binding protein-1c, and lipoprotein lipase	β-glucan	[1]

Abbreviations: 17β-HSD: 17β-hydroxysteroid dehydrogenase; ACC1: acetyl-CoA carboxylase 1; ACE: angiotensin converting enzyme; ACO: 1-aminocyclopropane-1-carboxylate oxidase; AGE: advanced glycation end products; AGPAT: 1-acylglycerol-3-phosphate O-acyltransferase 1; AgRP: agouti-related peptide; ALT: alanine transaminase; AMPK: 5′ adenosine monophosphate-activated protein kinase; Ang-I: angiotensin I; Ang-II: angiotensin II; AST: aspartate transaminase; AT-1: angiotensin II type 1 receptor; BMI: body mass index; BMR: basal metabolic rate; BUN: blood urea nitrogen; Ca: calcium; CAT: catalase; Cl: chloride; CNS: central nervous system; CPT1: carnitine Palmitoyltransferase 1A; CRE: creatinine; CRP: c-reactive protein; CVD: cardiovascular diseases; DGAT: diacylglycerol O-Acyltransferase 1; DPPH: 2,2-diphenyl-1-picrylhydrazyl; E1: estrone; E2: estradiol; FFA: free fatty acids; GPATR: glycerol-3-phosphate acyltransferase; GSH: glutathione; GSH-Px: glutathione peroxidase; HDL: high density lipoprotein; HFD: high fat diet; HMG-CoA: β-hydroxy, β methyl glutaryl COA; ICAM: Intercellular Adhesion Molecule; IGF1: IGF binding protein 1; IL-1: interleukin 1; IL-6: interleukin 6; K: potassium; LDL: low density lipoprotein; LPO: lipid peroxidation; MAPK: mitogen-activated protein kinases; MCH: melanin concentrating hormone; MDA: melondialdehyde; Mg: magnesium; mRNA: messenger ribonucleic acids; Na: sodium; NF-χB: nuclear factor kappa B; NO: nitric oxide; NPY: neuropeptide Y; OGTT: oral glucose tolerance test; P: phosphorus; POMC: pro-opiomelanocortin; PPAR-α: peroxisome proliferator-activated receptor alpha; PUFA: poly unsaturated fatty acids; RAS: renin-angiotensin-aldosterone system; ROS: reactive oxygen species; SCD1: stearoyl-CoA desaturase 1; SCFA: short chain fatty acids; SCFA: short chain fatty acids; SOD: superoxide dismutase; SREBP-1c: sterol regulatory element-binding transcription factor 1; STZ: streptozotocin; T-AOC: total antioxidant capacity; TBARS: thiobarbituric acid reactive substances; TC: total cholesterol; TG: triglycerides; TNF-α: tumour necrosis factor-α; UCP2: mitochondrial uncoupling proteins 2; UCP3: mitochondrial uncoupling proteins 3; VCAM: vascular cell adhesion molecule; VLDL: very low density lipoprotein; Δ4A: Δ4-androstenedione.

## Abbreviations

17β-HSD	17β-hydroxysteroid dehydrogenase
ACC1	acetyl-CoA carboxylase 1
ACE	angiotensin converting enzyme
ACO	1-aminocyclopropane-1-carboxylate oxidase
AGE	advanced glycation end products
AGPAT	1-acylglycerol-3-phosphate O-acyltransferase 1
AgRP	agouti-related peptide
ALT	alanine transaminase
AMPK	5′ adenosine monophosphate-activated protein kinase
Ang-I	angiotensin I
Ang-II	angiotensin II
AST	aspartate transaminase
AT-1	angiotensin II type 1 receptor
BMI	body mass index
BMR	basal metabolic rate
BUN	blood urea nitrogen
Ca	calcium
CAT	catalase
Cl	chloride
CNS	central nervous system
CPT1	carnitine Palmitoyltransferase 1A
CRE	creatinine
CRP	c-reactive protein
CVD	cardiovascular diseases
DGAT	diacylglycerol O-Acyltransferase 1
DPPH	2,2-diphenyl-1-picrylhydrazyl
E1	estrone
E2	estradiol
FFA	free fatty acids
GPATR	glycerol-3-phosphate acyltransferase
GSH	glutathione
GSH-Px	glutathione peroxidase
HDL	high density lipoprotein
HFD	high fat diet
HMG-CoA	β-hydroxy, β methyl glutaryl COA
ICAM	Intercellular Adhesion Molecule
IGF1	IGF binding protein 1
IL-1	interleukin 1
IL-6	interleukin 6
K	potassium
LDL	low density lipoprotein
LPO	lipid peroxidation
MAPK	mitogen-activated protein kinases
MCH	melanin concentrating hormone
MDA	melondialdehyde
Mg	magnesium
mRNA	messenger ribonucleic acids
Na	sodium
NF-χB	nuclear factor kappa B
NO	nitric oxide
NPY	neuropeptide Y
OGTT	oral glucose tolerance test
P	phosphorus
POMC	pro-opiomelanocortin
PPAR-α	peroxisome proliferator-activated receptor alpha
PUFA	poly unsaturated fatty acids
RAS	renin-angiotensin-aldosterone system
ROS	reactive oxygen species
SCD1	stearoyl-CoA desaturase 1
SCFA	short chain fatty acids
SCFA	short chain fatty acids
SOD	superoxide dismutase
SREBP-1c	sterol regulatory element-binding transcription factor 1
STZ	streptozotocin
T-AOC	total antioxidant capacity
TBARS	thiobarbituric acid reactive substances
TC	total cholesterol
TG	triglycerides
TNF-α	tumour necrosis factor-α
UCP2	mitochondrial uncoupling proteins 2
UCP3	mitochondrial uncoupling proteins 3
VCAM	vascular cell adhesion molecule
VLDL	very low density lipoprotein
WHO	world health organization
WLS	weight loss surgery
Δ4A	Δ4-androstenedione
